# Online cancer forums and complementary and alternative medicine in cancer care: effects of ehealth literacy and psychological distress on use and attitudes

**DOI:** 10.1007/s00520-026-11049-3

**Published:** 2026-08-04

**Authors:** G. Ciarlo, M. Oehlrich, J. Huebner

**Affiliations:** 1 Medical Clinic 1, Gastroenterology, Hepatology, Pneumology, Allergology, Nutritional Medicine, Endocrinology, Diabetology, University Hospital Frankfurt, Theodor-Stern-Kai 7, 60590 Frankfurt Am Main, Germany; 2 Management Department, Accadis Hochschule, Am Weidenring 4, 61352 Bad Homburg Vor Der Höhe, Germany; 3 Klinik Für Innere Medizin II, Universitätsklinikum Jena, Am Klinikum 1, 07747 Jena, Germany

**Keywords:** Online forums, Decision-making, CAM, Cancer care, eHealth literacy, Psychological distress

## Abstract

**Purpose:**

Online cancer forums are often assumed to promote misinformation and unsupported treatment claims, including some non-evidence-based complementary and alternative medicine (CAM) approaches. This study examined the relationship between online cancer forum use, attitudes toward complementary and alternative medicine (CAM), and the use of non-evidence-based therapies, with a specific focus on CAM approaches prevalent in German-speaking countries.

**Methods:**

A cross-sectional anonymous online survey was conducted in June–July 2025 among adult users of the largest German cancer forum, assessing forum use, CAM-related attitudes (defined according to the German S3 guideline checklist, version 1.2), eHealth literacy, psychological distress, and sociodemographic and disease-related variables. Forum use was dichotomized by median split, and data were analyzed using non-parametric tests and multivariable regression models.

**Results:**

Among 458 participants, high versus low forum use differed by sociodemographic and disease-related factors but was not associated with eHealth literacy or psychological distress. Among CAM users (44.3%, *n* = 203), CAM use intensity was moderate (median = 2, IQR 1–3) and showed no association with forum use, eHealth literacy, psychological distress, or sociodemographic variables; no independent predictors were identified. CAM-related forum discussions were rated as significantly less trustworthy than discussions on conventional cancer treatments (*t*(383) = 16.88, *p* < 0.001), independent of eHealth literacy, psychological distress, or participant role.

**Conclusion:**

The use of CAM approaches, including both evidence-based supportive interventions and non-evidence-based methods, was not associated with forum exposure, eHealth literacy, or psychological distress. Forum users demonstrated differentiated trust patterns, with consistently lower perceived credibility attributed to CAM-related discussions compared with discussions on conventional cancer treatments.

**Implications for cancer survivors:**

For cancer survivors, online cancer forums appear to function primarily as contextual support resources rather than drivers of non-evidence-based treatment decisions. Clinicians should therefore address forum use openly and early, focusing on guidance and shared decision-making instead of discouragement.

**Supplementary Information:**

The online version contains supplementary material available at 10.1007/s00520-026-11049-3.

## Introduction

In an increasingly digitized and fast-paced society, there is a growing need for quick and compact access to health-related information. At the same time, the availability of unfiltered, commercially biased, or medically unvalidated content carries the risk of influencing patient decision-making processes and promoting unrealistic expectations of physicians and therapeutic measures [1]. This can undermine the development of adequate eHealth literacy and at the same time impair patients' self-efficacy and successful shared decision-making practices in the doctor-patient relationship [2–4].

Studies have long examined the internet as a source of health-related information and have consistently underscored the substantial challenges and risks associated with its use. There is robust evidence that the internet has become an established and widely used tool for accessing health information [5, 6]; however, concerns about the quality, accuracy, and reliability of online content persist. In response, various initiatives have been developed to promote trustworthy digital health information and mitigate the spread of misinformation. These include the Health On the Net Foundation (HONcode), established in 1995, which certifies websites based on principles of transparency, authority, and evidence-based content [1, 7], as well as structured quality appraisal tools such as DISCERN, the Journal of the American Medical Association (JAMA) Benchmark Criteria, and more recently the Principles for Health-related Information on Social Media (PRHISM) [7–9].

Despite these efforts, recent evidence demonstrates that misinformation and potentially harmful statements remain highly prevalent in social media posts about cancer, with analyses indicating that approximately 27% of posts contain misinformation and 21% include harmful content [7]. Particularly in online forums, where medical information and unfiltered subjective experiences intersect, such content can contribute to misguided decisions and, in the worst case, to health-damaging behaviors—especially when these platforms are mistakenly perceived as indicators of one’s own eHealth literacy [9].

eHealth literacy is a central prerequisite for effective patient–provider interaction, as it shapes individuals’ capacity to understand, critically appraise, and apply medical information in clinical decision-making [10, 11]. Higher eHealth literacy is associated with more effective communication, including asking questions, clarifying uncertainties, and active participation in shared decision-making and treatment discussions, as well as greater involvement and satisfaction in medical decisions and self-management [12]. In contrast, limited eHealth literacy impairs comprehension of medical information, reduces engagement, and is often accompanied by shame or stigma, further restricting participation [10]. On the other hand, higher eHealth literacy is positively associated with confidence and trust in the healthcare system [13, 14], whereas insufficient eHealth literacy is linked to lower trust and increased psychological distress, particularly among individuals with chronic conditions [15].

A cancer diagnosis is often associated with considerable psychological stress and confronts patients with new emotional and cognitive challenges. Psychological distress can lead to avoidance behavior, intensive and often unstructured internet research, as well as increased vulnerability to misinformation [16, 17]. Under high stress, there is also an increased tendency to seek guidance in simplified explanatory models, promises of healing, or alternative treatment options [17]. In line with established coping models, those affected in such situations increasingly seek strategies that convey a sense of control, meaning, or emotional relief [18, 19]. Complementary and alternative medicine (CAM) encompasses a heterogeneous spectrum of approaches ranging from evidence-based supportive interventions, such as mindfulness-based therapies and yoga, to unproven or potentially harmful practices lacking scientific support. Digital platforms, and online forums in particular, contribute significantly to the dissemination of CAM-related content [20]; subjective testimonials shared there are often perceived as authentic and trustworthy and can influence attitudes toward CAM [20, 21]. Previous international and European studies have consistently shown that CAM use among cancer patients is associated with factors such as female gender, higher educational attainment, younger age, and greater desire for active participation in treatment decisions. Similar patterns have been reported in European populations, including Germany, Sweden, and Norway, where CAM use among cancer patients is widespread and primarily motivated by symptom management, quality-of-life improvement, and a desire for active participation in care [22–24].

The aim of this study is to systematically investigate the extent to which the use of online cancer forums is related to attitudes toward CAM, CAM use, and CAM-related decision-making. In addition, the role of eHealth literacy and psychological distress in this context will be analyzed. Specifically, the study examines how these two factors are associated with forum use, CAM-related attitudes, CAM use, and CAM-related decision-making. This should provide a better understanding of how digital information spaces influence patient decision-making processes and which individual characteristics increase susceptibility to unproven or potentially risky treatment options.

## Patients and methods

### Study population

**Fig. 1:**
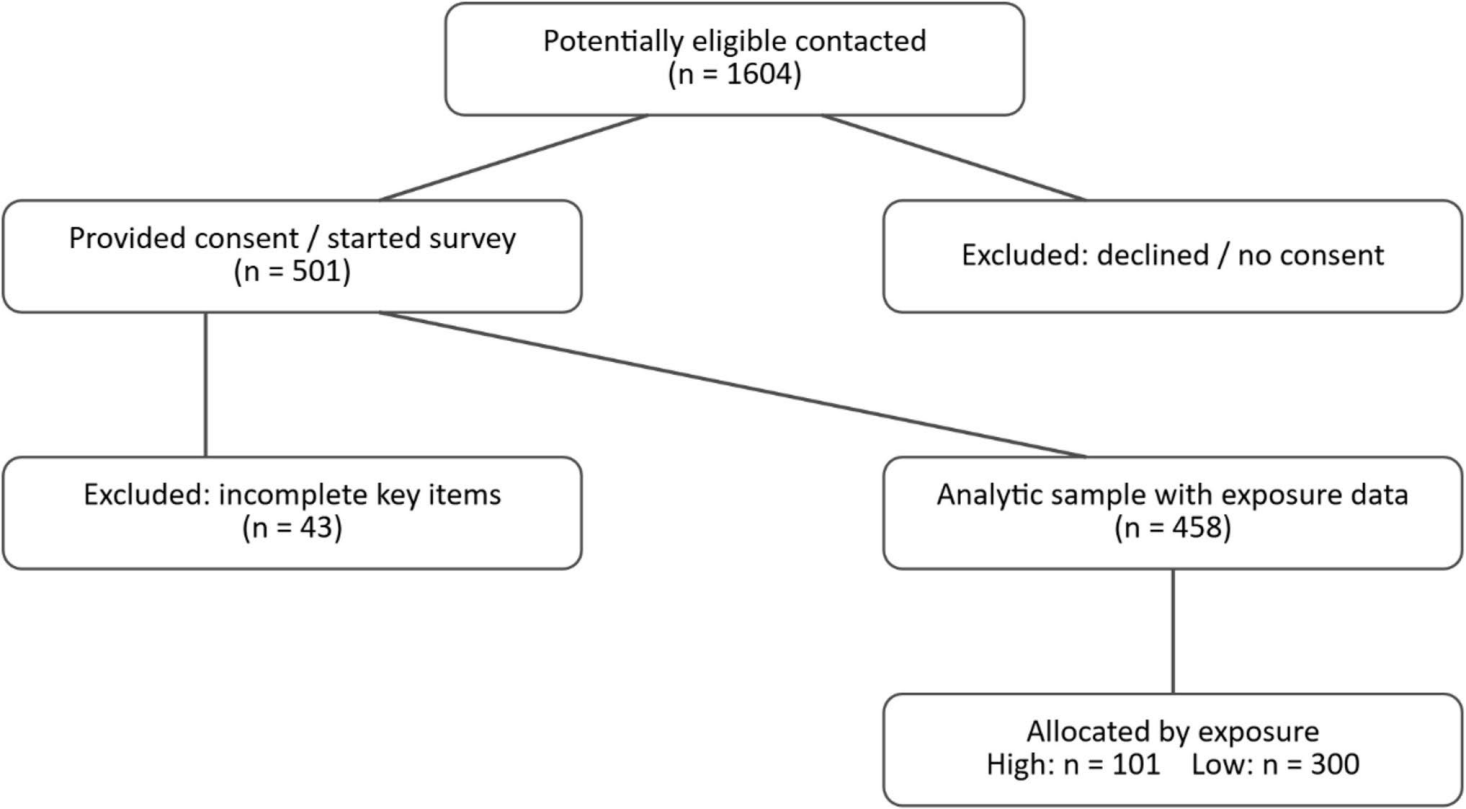
Flowchart of participant inclusion and group allocation. Note. Flow diagram showing the number of potentially eligible Krebs-Kompass users contacted, the number excluded due to non-consent, and the final analytic sample. Participants were categorized into high versus low forum exposure groups based on their self-reported visit frequency, dichotomized at the sample median

This cross-sectional study employed an anonymous online survey conducted between June and July 2025 among users of Krebs-Kompass (www.krebs-kompass.de), a peer support forum for individuals affected by cancer in German-speaking countries. The survey was administered via the Qualtrics platform. Registered Krebs-Kompass users were eligible for invitation if they had either newly registered or logged into their account at least once between January 1, 2024, and the end of the recruitment period, regardless of whether they had actively posted in the forum. Based on these criteria, 1604 registered users were contacted via email and invited to participate in the survey. Two reminder emails were sent during the recruitment period. In addition, the study invitation was displayed publicly on the Krebs-Kompass homepage and as a pinned announcement across all forum sections, allowing participation by both registered and non-registered visitors. Participants received study information and provided electronic informed consent prior to participation. Of 501 fully completed questionnaires, 458 were included in the analysis; 43 were excluded due to missing consent. Recruitment via the platform ensured prior exposure to online cancer forums. The final sample predominantly resided in German-speaking countries. The manuscript was revised in consideration of the Strengthening the Reporting of Observational Studies in Epidemiology (STROBE) recommendations for cross-sectional studies [25]. The participant flow and study inclusion process are summarized in Fig. 1.

### Inclusion and exclusion criteria

Eligible participants were individuals aged 18 years or older who identified as cancer patients, relatives, or friends and had accessed the forum at least once, either through registration, login activity, or visiting the Krebs-Kompass website during the recruitment period. Active participation in forum discussions (e.g., posting or commenting) was not required. Participants were required to provide electronic informed consent before beginning the anonymous survey. Individuals were excluded if they declined consent.

### Endpoints

The primary endpoint of this study was to examine the association between online cancer forum use, CAM-related attitudes, CAM use, and CAM-related decision-making, while exploring the potential roles of eHealth literacy and psychological distress in these relationships.

**Fig. 2:**
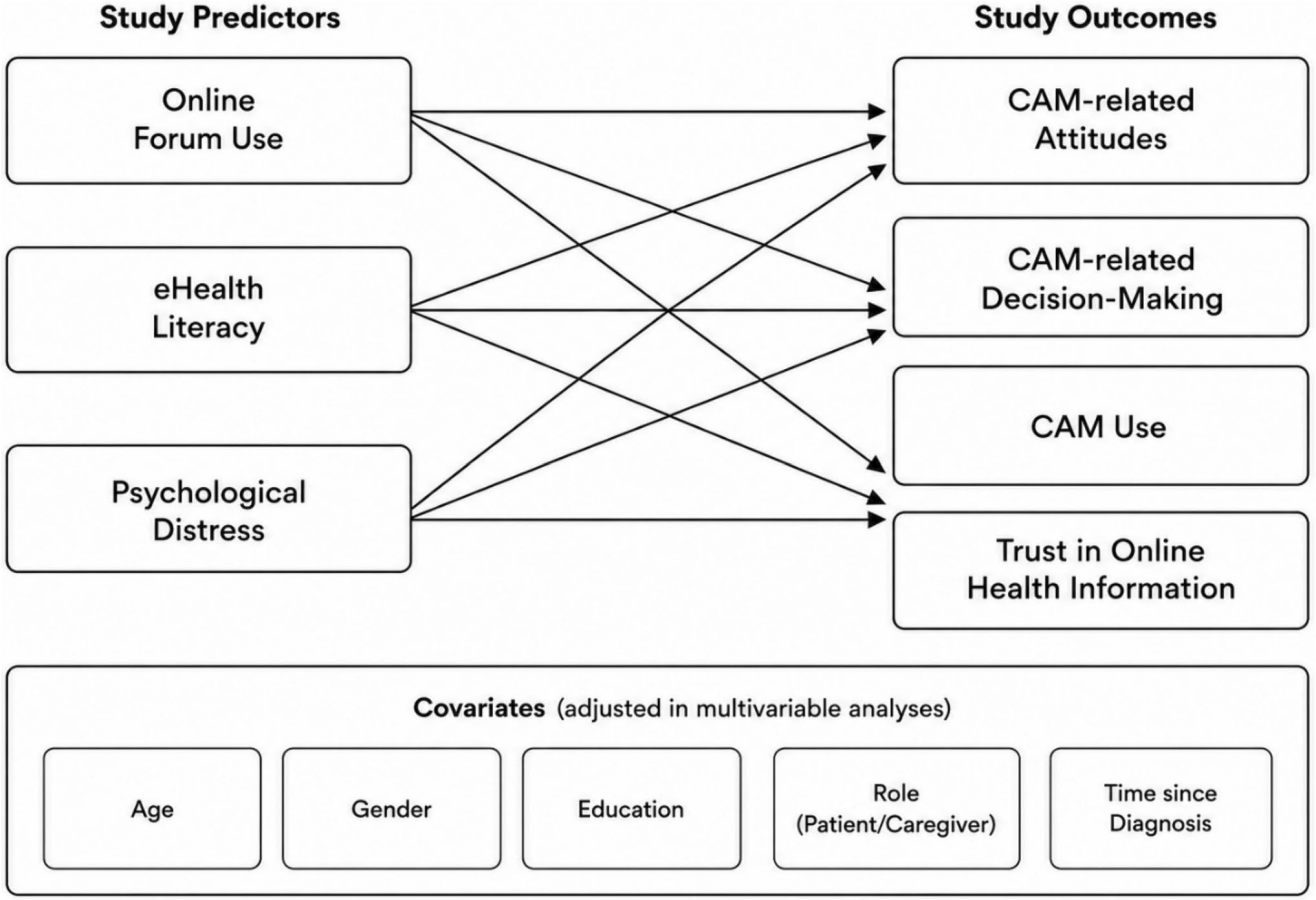
Conceptual framework of the study. Note. The relationships between the investigated predictors, outcomes, and covariates are summarized in this figure

Secondary endpoints included examining associations between eHealth literacy, psychological distress, online forum engagement, perceived trustworthiness of online health information, exposure to non-evidence-based content, and CAM-related attitudes and decision-making. The conceptual framework underlying these relationships is illustrated in Fig. 2.

### Questionnaire

A standardized, self-administered online questionnaire was developed to address the objectives of the present study. The instrument consisted of 24 core items across four thematic domains. All responses were recorded anonymously.

Prior to fielding, the questionnaire underwent a technical pilot test in Qualtrics (*n* = 12) and two cognitive interviews with cancer patients to assess clarity, item order, and response options. Feedback resulted in minor wording revisions, removal of redundant CAM items, and the addition of “don’t know” response categories for selected clinical variables. Participants involved in cognitive interviewing and pilot testing were excluded from the final data analysis.

The questionnaire covered the following four domains:

- Sociodemographic characteristics and online eHealth literacy: Participants reported age, gender, and educational attainment. eHealth literacy was assessed using the eight-item eHealth Literacy Scale (eHEALS), German version, which measures perceived competencies in locating, evaluating, and applying online health information [26]. In the original validation study, the scale demonstrated good internal consistency, with Cronbach’s *α* = 0.88 for the information-seeking subscale and Cronbach’s *α* = 0.83 for the information-appraisal subscale.
- Disease-related information and psychological distress: Respondents indicated their role in relation to the cancer diagnosis (patient, survivor, relative, or friend), cancer type, disease stage, year of diagnosis, and treatments received in the previous three months. Psychological distress over the past 2 weeks was assessed using the Patient Health Questionnaire-4 (PHQ-4), an ultra-short screening tool combining the Patient Health Questionnaire-2 (PHQ-2) depression scale and the Generalized Anxiety Disorder-2 (GAD-2) anxiety scale, with demonstrated reliability and validity in both the original and German-language versions [27, 28].
- Online forum use and engagement: Forum exposure was measured via self-reported visit frequency, typical duration per visit, participation style (active posting versus passive reading), motivations for forum use, and criteria influencing trust in treatment recommendations from other users. Visit frequency served as the primary exposure variable and was dichotomized using a median split to facilitate group-based comparisons and multivariable modeling, given the non-normal distribution and unequal category frequencies. Visit frequency showed only minimal correlation with session length (Spearman *ρ* = 0.07, *p* = 0.201), suggesting that frequency and session duration may reflect distinct dimensions of forum engagement. Participants additionally reported the types of online cancer-related websites they used (multiple responses allowed), including patient forums, official medical websites, CAM-related websites, and media-based platforms.
- Attitudes toward CAM: Attitudes toward CAM were assessed using an adapted checklist derived from the German Oncology Guideline Program and aligned with the evidence- and consensus-based S3 guideline on complementary medicine in oncology [29, 30]. The original checklist was modified by converting categorical response options into Likert-scale ratings to assess perceived effectiveness and trustworthiness of CAM-related approaches and by reducing overlapping CAM categories to improve questionnaire usability and comparability with previous international survey studies [31]. Participants rated the perceived effectiveness of a range of CAM procedures, including methods supported by varying levels of evidence as well as unproven treatments. CAM interventions were classified according to the S3 guideline on complementary medicine in oncology [30] and the categories of the National Center for Complementary and Integrative Health (NCCIH) [32]. Additional items assessed perceived trustworthiness of CAM-related forum discussions, confidence in distinguishing CAM from conventional medicine, exposure to non-evidence-based treatment discussions, and the perceived influence of such discussions on CAM-related decision-making.

For analytical purposes, questionnaire variables were categorized as predictors, outcomes, or covariates according to their role in the analyses. The main outcomes included CAM-related attitudes, CAM use, trust in online health information, and the perceived influence of forum discussions on decisions regarding non-evidence-based treatments. eHealth literacy (eHEALS) and psychological distress (PHQ-4) were examined as key explanatory variables, while sociodemographic and disease-related characteristics were included as potential covariates. Missing values were reported descriptively as “no answer” categories where appropriate and handled by listwise deletion in multivariable analyses. The full questionnaire is provided as Supplementary Material.

### Statistical analysis

Statistical analyses were performed using IBM SPSS Statistics for Windows, Version 31.0 (IBM Corp., Armonk, NY, USA). Descriptive statistics were used to characterize the study sample and included frequencies and percentages for categorical variables as well as means, standard deviations, medians, and interquartile ranges for continuous variables, as appropriate. Group differences were examined using chi-square or Fisher’s exact tests for categorical variables and non-parametric tests for continuous variables. Where applicable, standardized mean differences were calculated to quantify effect sizes.

Internal consistency of multi-item scales was assessed using Cronbach’s alpha. eHealth literacy was operationalized as a composite score calculated as the mean of eight items (Cronbach’s *α* = 0.90), and psychological distress was operationalized as a sum score of four items assessing depressive and anxiety-related symptoms (Cronbach’s *α* = 0.92).

Because the questionnaire did not include an explicit response option indicating non-use of complementary and alternative medicine (CAM), CAM use was operationalized as the number of different CAM modalities applied. Analyses of CAM use were therefore restricted to respondents reporting CAM use and focused on patterns and intensity of use. Given the right-skewed distribution of the CAM use count variable, non-parametric methods were applied for bivariate analyses. Associations between CAM use intensity and continuous variables, including eHealth literacy and psychological distress, were assessed using Spearman rank correlations, while group differences were examined using Mann–Whitney *U* or Kruskal–Wallis tests.

Trust in online forum discussions was examined by comparing trust ratings for CAM-related versus conventional cancer treatment discussions using paired-sample analyses. A trust difference score was calculated and used in subsequent correlation analyses, group comparisons, and multivariable linear regression models.

Multivariable analyses were conducted using linear and logistic regression models with covariates selected based on prior literature on CAM use, online health information-seeking behavior, and psychological distress in cancer populations [12, 15,17,16 –], as well as an a priori conceptual framework. Predictors included forum use, age, gender, educational level, role (patient vs. non-patient), time since diagnosis, eHealth literacy, and psychological distress. Logistic regression models were used to examine predictors of high versus low forum exposure and strong perceived influence of online forum discussions on CAM-related decision-making. Linear regression models were applied to analyze continuous outcomes, including CAM use intensity and trust difference scores between CAM-related and conventional treatment discussions. Given the exploratory nature of the analyses and the cross-sectional study design, regression results were interpreted cautiously.

### Ethical vote

The data used in this study were collected in Germany as part of an anonymous online survey con-ducted independently from the Harvard T.H. Chan School of Public Health. Under applicable German regulations, ethics committee review is not required for studies of this type when conducted outside a university or medical chamber context. The survey was carried out in accordance with relevant German data protection laws, and informed consent was obtained electronically prior to participation. All data were provided to the authors in fully anonymized form by the forum administrator. No identifiers or indirect identifiers were collected, and re-identification of individual participants is not possible. Consistent with U.S. federal regulations (45 CFR 46.104[d][2][i]), such fully anonymized, minimal-risk survey research is not considered human subjects research requiring Institutional Review Board (IRB) review.

## Results

### Demographic data

Overall, between June and July 2025, 458 participants were included in the study. The largest proportion of participants was between 46 and 65 years of age. The sample consisted of 67% females and 30% males. Sociodemographic and clinical characteristics stratified by forum exposure (high vs. low) are presented in Table 1. While age, gender, education, cancer type, and disease stage were largely comparable between exposure groups (all SMDs ≤ 0.23), notable differences emerged in participants’ roles and time since diagnosis. Individuals with high forum exposure were more frequently family members or partners of patients (36% vs. 19%), whereas survivors were substantially more common in the low‑exposure group (52% vs. 30%). Moreover, high‑exposure users were more often recently diagnosed (< 1 year, 37% vs. 22%), while long‑term survivors (> 10 years) were predominantly represented in the low‑exposure group (25% vs. 11%). Treatment patterns in the past three months were largely comparable between exposure groups, with no clinically meaningful differences across conventional or complementary therapies (all SMDs ≤ 0.23). Combination therapy referred to the concurrent use of multiple listed modalities, whereas “other” treatments—e.g., antibodies or radioiodine therapy—were more frequent in the low/non-exposure group.

**Table 1:** Sociodemographic and clinical characteristics by cancer forum usage (high vs. low exposure)

Characteristic	Overall N	High exposure (*n* = 101)	Low exposure (*n* = 300)	SMD	*p*-value
*Age (years)*	*401*			*0.23*	*0.709*
18–25		0 (0%)	2 (0.7%)		
26–35		5 (5%)	19 (6.3%)		
36–45		12 (11.9%)	48 (16%)		
46–55		34 (33.7%)	81 (27%)		
56–65		34 (33.7%)	93 (31%)		
66–75		15 (14.9%)	51 (17%)		
76+		1 (1%)	6 (2%)		
*Gender*	*400*			*0.18*	*0.069*
Male		24 (23.8%)	100 (33.4%)		
Female		77 (76.2%)	199 (66.6%)		
Missing value		0	1		
*Education*	*401*			*0.02*	*0.633*
Vocational or academic degree		83 (82.2%)	244 (81.3%)		
Middle school diploma		18 (17.8%)	51 (17%)		
No school diploma or other		0 (0%)	1 (0.3%)		
Other^1^		0 (0%)	4 (1.3%)		
*Role*	*400*			*0.35*	*<0.001*
Currently in treatment		31 (30.7%)	72 (24%)		
Survivor		30 (29.7%)	155 (51.7%)		
Family member/partner		36 (35.6%)	58 (19.3%)		
Close friend		0 (0%)	2 (0.7%)		
Other^2^		4 (4%)	12 (4%)		
Missing value		0	1		
*Cancer type*	*396*			*0.17*	*0.278*
Bladder		2 (2%)	3 (1%)		
Breast		27 (27%)	86 (29.1%)		
Colorectal		4 (4%)	3 (1%)		
Leukemia/lymphoma		3 (3%)	15 (5.1%)		
Lung		13 (12.9%)	25 (8.4%)		
Pancreatic		1 (1%)	6 (2%)		
Prostate		3 (3%)	4 (1.4%)		
Skin		6 (6%)	11 (3.7%)		
Other^3^		40 (39.6%)	142 (48%)		
Don´t know		1 (15%)	1 (0.3%)		
Missing value		1	4		
*Stage*	*376*			*0.21*	*0.062*
Localized		37 (37.4%)	131 (47.3%)		
Regional		21 (21.2%)	56 (20.2%)		
Metastatic		32 (32.3%)	55 (19.9%)		
Don’t know		9 (9.1%)	35 (12.6%)		
Missing value		2	23		
*Treatment in the last 3 months*	*353*			*0.23*	*0.042*
Surgery		7 (7.2%)	22 (8.6%)		
Chemotherapy		3 (3.1%)	8 (3.1%)		
Radiotherapy		0 (0%)	5 (2%)		
Immunotherapy		2 (2.1%)	10 (3.9%)		
Endocrine therapy		4 (4.1%)	14 (5.5%)		
Experimental therapies (e.g., studies)		2 (2.1%)	0 (0%)		
Palliative care		4 (4.1%)	4 (1.6%)		
CAM		0 (0%)	4 (1.6%)		
Other^4^		20 (20.6%)	83 (32.4%)		
Combination therapy		51 (52.6%)	99 (38.7%)		
Don´t know		4 (4.1%)	7 (2.7%)		
No answer		4	44		
*Time since diagnosis*	*396*			*0.27*	*0.006*
<1 year		37 (36.6%)	65 (22%)		
1–2 years		23 (22.8%)	81 (27.5%)		
2–5 years		21 (20.8%)	52 (17.6%)		
5–10 years		9 (8.9%)	23 (7.8%)		
>10 years		11 (10.9%)	74 (25.1%)		
Missing value		0	5		

Values are shown as *n* (%). Standardized mean differences (SMD) and p-values from fisher’s exact test or chi-squared test with simulation (9999 replicates) are reported. Percentage may not sum to 100% due to rounding. ^1^Other= not further differentiated. ^2^Other= bereaved; general interest; both patient and relative; unconfirmed diagnosis; elevated familial risk. ^3^Other= GIT (esophagus, stomach); liver/biliary; endocrine (NET, thyroid); gynecologic (cervix, ovary, uterus, vulva, endometrium); kidney; testis; bone/cartilage; ENT; brain. ^4^Other= antibodies, radioiodine therapy, *transarterial chemoembolization (TACE)*, CyberKnife, preventive examinations, surveillance, none

### Patterns and correlates of online forum use among individuals affected by cancer

From 458 participants, 401 provided valid information on their use of online forums on the topic of cancer. The majority used forums rarely (39%) or sometimes (30%), while 13% visited them very frequently and 13% frequently. The mean forum use frequency score was 3.12 on a five-point scale (SD = 1.11). After dichotomization, 75% (*n* = 300) were classified as low exposure and 25% (*n* = 101) as high exposure. High-frequency forum use varied significantly across the course of cancer survivorship. Participants diagnosed within the past year reported the highest rates of intensive forum use, whereas long-term survivors (> 10 years since diagnosis) showed substantially lower exposure (Table 1; Supplementary Fig. S1). Among participants providing valid responses (*n* = 242), patient forums and self-help groups were by far the most frequently reported primary source of online cancer-related information (71%). Other website types, including media-based platforms (9%), combinations of multiple website types (13.6%), official medical websites (1%), and CAM-specific websites (0.4%), were used considerably less frequently. The distribution of website types did not differ significantly between participants with high versus low use of the Krebs-Kompass forum. There were no significant correlations between high/low exposure and age (*χ*^2^(6) = 3.76, *p* = 0.709), gender (*χ*^2^(1) = 3.31, *p* = 0.069), or educational attainment (*χ*^2^(3) = 1.72, *p* = 0.633). However, women tended to have a higher proportion of high exposure (28% vs. 20% for men).

A significant correlation was found for the category of those affected (*χ*^2^ (4) = 18.49, *p* < 0.001): relatives/partners had the highest proportion of high exposure (38%), followed by patients undergoing therapy (30%) and “others” (24%), while former patients had significantly lower high exposure rates (16%). The type of cancer was not significantly associated (*χ*^2^(9) = 10.96, *p* = 0.278), nor was the stage of cancer (*χ*^2^ (3) = 7.32, *p* = 0.062), although a higher proportion of high exposure was seen in metastatic diseases (37%).

The time of diagnosis showed a significant correlation (*χ*^2^(4) = 14.44, *p* = 0.006): People diagnosed less than 1 year ago reported high exposure most frequently (36%), while the proportion was significantly lower for diagnoses made more than 10 years ago (13%). Therapy in the last three months was also significant (*χ*^2^(10) = 18.84, *p* = 0.042), with higher high exposure rates for more intensive forms of therapy.

Logistic regression (*n* = 373) yielded a non-significant overall model (*χ*^2^(7) = 13.40, *p* = 0.063; Nagelkerke *R*^2^ = 0.051). Only the time of diagnosis was a significant predictor (*B* =  − 0.234, *p* = 0.008; OR = 0.79), meaning that a more recent diagnosis increased the probability of high exposure. All other predictors (age, gender, education, category of affected person, type of cancer, stage of cancer) showed no significant effects.

### Influence of online cancer forums on CAM-related attitudes and decision-making

Depending on the variable, there were between 381 and 386 valid cases. The trustworthiness of online discussions on complementary and alternative medicine (CAM) practices was predominantly rated as neutral (46%), followed by somewhat uncertain (26%) and not at all certain (15%); only 6% rated them as somewhat reliable or very reliable. Discussions on conventional cancer treatments were rated much more positively: 50% rated them as somewhat reliable and 5% as very reliable, while 34% responded neutrally and 9% responded as uncertain or not at all reliable. Confidence in the accuracy of CAM information was predominantly neutral (37%), somewhat uncertain (22%), or not at all confident (16%). Posts about non-evidence-based treatments were predominantly rarely (40%) or never (32%) read in the last 3 months; 18% reported “sometimes,” 7% “frequently,” and 2% “very frequently.” The willingness to consider non-evidence-based treatments was predominantly low (33%) or non-existent (38%); 23% stated “moderate,” 5% “strong,” and 0.5% “very strong.”

The cross-tabulations showed no differences between high- and low-exposure groups in terms of the trustworthiness of CAM discussions (e.g., neutral, 33% high vs. 47% low; somewhat uncertain, 27% vs. 26%; not at all certain, 16% vs. 14%). The linear-by-linear association was not significant (*χ*^2^ = 0.430, *p* = 0.512). There were also no group-specific differences in the trustworthiness of conventional methods (e.g., rather confident, 27% high vs. 73% low; neutral, 23% vs. 77%); and the linear-by-linear association was also not significant (*χ*^2^ = 0.760, *p* = 0.383). The same applied to certainty regarding the accuracy of CAM information (neutral, 27% high vs. 73% low; rather uncertain, 25% vs. 75%); the linear-by-linear association was not significant (*χ*^2^ = 2.027, *p* = 0.155).

The percentage distributions of the high-exposure group are shown in Table 2. A highly significant linear trend was found for reading non-evidence-based treatments (linear-by-linear association: *χ*^2^ = 46.131, *p* < 0.001). There was also a significant linear correlation for the willingness to consider non-evidence-based treatments (*χ*^2^ = 46.131, *p* < 0.001). There were no significant linear trends for all other variables.

**Table 2:** High exposure percentages for all ordinal measures related online cancer forum use

Variable	Response category	High exposure (%)
**Trust in discussions on CAM effectiveness and safety**	Very certain1	100
	Rather certain	33.3
	Neutral	24.3
	Rather uncertain	26.3
	Not certain at all	28.6
	Don’t know	17.9
**Trust in conventional treatments**	Very certain	33.3
	Rather certain	26.8
	Neutral	23.4
	Rather uncertain	29.2
	Not certain at all	25.0
	Don’t know	16.7
**Certainty about correctness of CAM information**	Very certain	44.4
	Rather certain	27.9
	Neutral	26.6
	Rather uncertain	24.7
	Not certain at all	18.0
	Don’t know	26.3
**Reading non-evidence-based treatments**	Very frequently1	100
	Frequently	51.9
	Sometimes	36.2
	Rarely	22.7
	Never	12.3
**Willingness to consider non-evidence-based treatments**	Very strong	50.0
	Strong	35.0
	Moderate	30.4
	Slight	26.6
	Not at all	22.4

^1^Cell count extremely small (*n* ≤ 9); percentages should be interpreted with caution

### Patterns of online cancer forum use in relation to eHealth literacy and psychological distress

The eHealth literacy scale demonstrated high internal consistency (Cronbach’s *α* = 0.90). Overall, participants reported a high level of eHealth literacy (M = 4.25, SD = 0.61), with particularly strong agreement regarding the ability to critically evaluate online health information and distinguish reliable from unreliable sources. Psychological distress scores indicated a relevant burden, with a mean score of 8.23 (SD = 3.48). Detailed item-level descriptive statistics for both scales are provided in Supplementary Tables S1 and S2. When comparing high-frequency users of online cancer forums with low or non-users, no significant differences in eHealth literacy were observed (Mann–Whitney *U* = 15 679, Z = 0.58, *p* = 0.56, *r* = 0.03). However, high-frequency forum users reported significantly higher levels of psychological distress than low or non-users (Mann–Whitney *U* = 17 323, Z = 2.52, *p* = 0.012, *r* = 0.13).

Spearman correlation analyses supported these findings. Forum use was not associated with eHealth literacy (*ρ* = 0.03, *p* = 0.56), whereas psychological distress showed a small but significant positive correlation with intensive forum use (*ρ* = 0.13, *p* = 0.012). No significant correlation was found between eHealth literacy and psychological distress (*ρ* =  − 0.07, *p* = 0.16).

In a multivariable logistic regression model adjusting for educational level and time since diagnosis, eHealth literacy was not independently associated with high-frequency forum use (OR = 0.97, 95% CI 0.66–1.41, *p* = 0.86). Psychological distress showed a trend toward an independent association (OR = 1.07, 95% CI 1.00–1.14, *p* = 0.056). Shorter time since diagnosis was significantly associated with higher odds of intensive forum use (OR = 0.81, 95% CI 0.69–0.96, *p* = 0.016).

### eHealth literacy and psychological distress in relation to CAM-related attitudes and decision-making

Participants reported a generally cautious stance toward CAM-related information in online cancer forums. Trust in CAM-related forum discussions and confidence in the correctness of CAM-related information were predominantly moderate to low (median = 3), whereas exposure to non-evidence-based treatment content was infrequent and the perceived influence of forum discussions on considering such treatments was low (median = 4).

Spearman correlation analyses revealed no associations between eHealth literacy and trust in CAM-related forum content or perceived correctness of information (*ρ* between − 0.05 and 0.06, all *p* > 0.10). Higher eHealth literacy was, however, weakly associated with a stronger perceived influence of forum discussions on decision-making regarding non-evidence-based treatments (*ρ* = 0.14, *p* = 0.005). Psychological distress showed a more consistent pattern, with higher distress associated with more frequent exposure to non-evidence-based treatment content (*ρ* =  − 0.22, p < 0.001) and with stronger perceived influence of forum discussions on treatment considerations (*ρ* =  − 0.18, *p* < 0.001), while no associations with trust or perceived correctness were observed.

Group comparisons supported these findings. Participants reporting strong trust in CAM-related information exhibited higher psychological distress than those reporting lower trust (p = 0.021), whereas eHealth literacy did not differ between groups. Individuals endorsing CAM primarily as a complementary rather than alternative approach demonstrated higher eHealth literacy (*p* = 0.009) without differences in psychological distress. Strong perceived influence of online forum discussions on CAM-related decision-making was associated with higher distress levels (*p* = 0.020), but not with eHealth literacy.

In a multivariable logistic regression model predicting strong influence of online forum discussions on CAM-related decision-making, psychological distress showed a trend toward an independent association (OR = 1.09, 95% CI approximately 0.99–1.20, *p* = 0.073), whereas eHealth literacy was not independently associated with the outcome (*p* = 0.88). Educational level and time since diagnosis were not significant predictors, and overall model fit was low (Nagelkerke *R*^2^ = 0.047).

### Actual use of complementary and alternative medicine in cancer care

Of the total sample, 203 participants (44.3%) provided valid responses to the items assessing the use of CAM. All subsequent analyses of CAM use were restricted to this subgroup. Because the questionnaire did not include an explicit response option indicating non-use of CAM, CAM use was operationalized as the number of different CAM modalities applied by each respondent.

Among CAM users, the number of reported CAM modalities ranged from 1 to 9. The median number of applied methods was 2 (interquartile range 1–3), with a mean of 2.04 modalities (SD = 1.34), indicating a generally moderate intensity of CAM use with considerable interindividual variability. The distribution of CAM use intensity was markedly right-skewed.

**Fig. 3:**
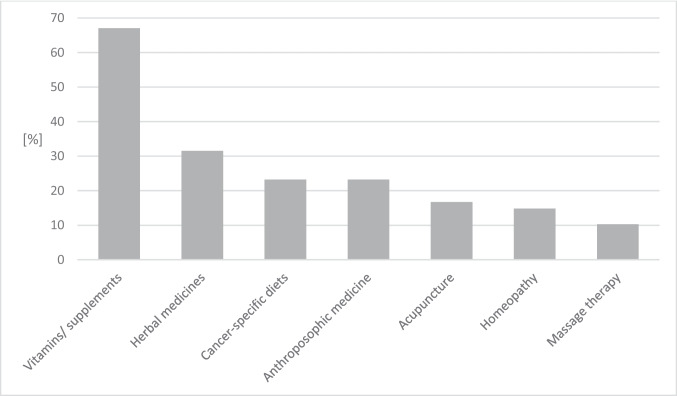
Distribution of reported CAM Methods. Note. Antroposophic medicine including misteltoe therapy

With regard to specific CAM modalities, vitamins and dietary supplements (e.g., vitamins and minerals) were the most frequently reported approaches, followed by herbal remedies. The full distribution of reported CAM modalities is shown in Fig. 3, illustrating that other CAM approaches were used less frequently, whereas the use of more controversial alternative treatment concepts was rare.

Bivariate analyses revealed no significant association between the number of applied CAM modalities and eHealth literacy (Spearman’s *ρ* = 0.06, *p* = 0.404). Similarly, psychological distress was not significantly correlated with CAM use intensity (*ρ* =  − 0.01, *p* = 0.894). No significant relationship was observed between eHealth literacy and psychological distress themselves (*ρ* =  − 0.07, *p* = 0.160).

Non-parametric group comparisons showed no significant differences in the number of applied CAM modalities between men and women (Mann–Whitney *U* = 3948.5, *p* = 0.228). Likewise, participants with high versus low use of online cancer forums did not differ significantly in their CAM use intensity (Mann–Whitney *U* = 3742.0, *p* = 0.688).

In a multiple linear regression model including eHealth literacy, psychological distress, gender, educational level, time since cancer diagnosis, and online forum use, the overall model was not statistically significant (*F* = 0.47, *p* = 0.830) and explained only a negligible proportion of variance in the number of applied CAM modalities (adjusted *R*^2^ =  − 0.017). None of the included predictors showed a significant independent association with CAM use intensity.

### Perceived effectiveness of specific CAM modalities

Participants rated discussions in online forums about the effectiveness and safety of complementary and alternative medicine (CAM) as significantly less trustworthy than discussions on conventional cancer treatments (M = 3.72, SD = 1.04 vs. M = 2.61, SD = 0.98), *t*(383) = 16.88, *p* < 0.001, with a large standardized mean difference (dz = 0.86, 95% CI [0.74, 0.98]). Despite this pronounced difference in perceived trustworthiness, no significant associations were observed between trust in CAM-related forum discussions and the perceived effectiveness of specific CAM modalities, including homoeopathy, cancer diets, anthroposophic medicine, massage therapies, and German New Medicine (all *χ*^2^ tests, *p* > 0.30).

Perceived effectiveness of individual CAM modalities was also unrelated to participants’ self-reported confidence in distinguishing between conventional medical treatments and CAM approaches (all *χ*^2^ tests, *p* > 0.50). Logistic regression analyses further supported these findings: neither eHealth literacy, perceived trustworthiness of CAM-related forum discussions or conventional treatment discussions, perceived correctness of CAM-related information, nor exposure to non-evidence-based treatment content significantly predicted endorsement of homoeopathy (*χ*^2^(5) = 7.88, *p* = 0.163; Nagelkerke *R*^2^ = 0.075) or cancer diets (*χ*^2^(5) = 3.24, *p* = 0.662; Nagelkerke *R*^2^ = 0.027) as effective. Across models, explained variance was low and classification accuracy did not exceed baseline levels.

Taken together, the results indicate that although participants clearly differentiated between CAM-related and conventional forum discussions in terms of perceived trustworthiness, this distinction did not translate into systematic differences in the perceived effectiveness of specific CAM modalities.

### Trust in CAM-related versus conventional treatment discussions in online forums

The difference score reflecting trust in CAM-related versus conventional treatment discussions was positive on average, indicating lower trust in CAM-related discussions. Across 384 participants with complete data, values ranged from − 4 to + 4, with a mean of 1.11 (SD = 1.29), reflecting substantial interindividual variability alongside a clear overall preference for conventional treatment discussions.

The trust difference was not significantly associated with eHealth literacy (Spearman’s *ρ* = 0.07, *p* = 0.154) or psychological distress (*ρ* =  − 0.06, *p* = 0.279). Thus, neither informational competence nor emotional burden was related to the magnitude of trust differentiation between CAM-related and conventional discussions.

Participants with high versus low frequency of online cancer forum use did not differ in their trust difference scores (Mann–Whitney *U* = 13,973, *p* = 0.863), indicating that forum exposure was not associated with stronger or weaker differentiation. Consistent with these findings, a multiple linear regression model including eHealth literacy, psychological distress, educational level, and forum use frequency was not statistically significant (*F*(4,369) = 1.88, *p* = 0.113) and explained only a small proportion of variance (*R*^2^ = 0.020; adjusted *R*^2^ = 0.009). None of the individual predictors reached statistical significance, although psychological distress showed a non-significant trend toward smaller trust differences (*B* =  − 0.03, *p* = 0.093).

Overall, trust in CAM-related discussions was consistently lower than trust in discussions on conventional cancer treatments, and this trust gap remained stable across levels of eHealth literacy, psychological distress, educational background, and online forum use.

### Role differences in online forum use and CAM-related attitudes

Participants’ role in relation to the cancer diagnosis was associated with differences in online forum use but not with CAM-related attitudes. Patients reported a significantly higher frequency of online cancer forum use than non-patients (Mann–Whitney *U* = 18,797.5, *p* = 0.011). In contrast, no significant role differences were observed regarding trust in online forum discussions. Patients and non-patients (e.g., relatives and partners) did not differ in their perceived trustworthiness of CAM-related discussions (*p* = 0.205) or of discussions addressing conventional cancer treatments (*p* = 0.789). Accordingly, the trust difference score reflecting relative trust in CAM versus conventional discussions also did not differ significantly between groups (Mann–Whitney *U* = 15,746.0, *p* = 0.323).

No role-based differences emerged for further CAM-related attitudes. Patients and non-patients reported comparable confidence in distinguishing between conventional medical treatments and CAM approaches (*p* = 0.876) and did not differ in their perceived correctness of CAM-related information provided in online forums (*p* = 9.168). Likewise, the reported influence of forum discussions on the willingness to consider or recommend non-evidence-based treatments was similar in both groups (*p* = 0.105).

These findings were supported by multivariate analyses. A linear regression model including role (patient vs. non-patient), eHealth literacy, psychological distress, and frequency of forum use did not significantly predict trust in CAM-related forum discussions (*F*(4,370) = 0.67, *p* = 0.611; adjusted *R*^2^ =  − 0.004), and none of the predictors showed a significant independent association.

Overall, patients differed from non-patients primarily in their frequency of online forum use, whereas trust in forum-based discussions and CAM-related attitudes were comparable across roles.

## Discussion

The present study found no statistically significant association between online forum use, eHealth literacy, psychological distress, and the intensity of CAM use among users of a moderated online cancer forum. Although psychological distress was weakly associated with more intensive forum use and greater perceived influence of forum discussions on CAM-related decision-making, participants generally reported differentiated trust judgments, with CAM-related discussions consistently perceived as less trustworthy than discussions on conventional cancer treatments. Rather, previous research indicates that these factors are more closely related to subjective information needs and the desire to participate actively in health-related decision-making processes [33]. At the same time, empirical studies have shown that increased psychological distress or depressive symptoms can impair the ability to consistently translate existing knowledge into health-promoting behavior [34], particularly in the context of complex decisions under uncertainty and the evaluation of competing information sources [35]. From this perspective, the use of unconventional information or therapy options may reflect attempts to restore perceived control, self-efficacy, or a sense of agency, as suggested in prior theoretical and empirical literature [18, 19].

Against this theoretical background, the absence of direct associations between eHealth literacy, psychological distress, and the intensity of CAM use observed in the present study suggests that these factors are more likely to shape attitudes, perceptions, and informational orientations than actual usage behavior. In other words, while eHealth literacy and psychological distress may influence how information is evaluated and what role patients wish to assume in decision-making, they do not appear to directly translate into increased engagement with CAM.

These findings align with current clinical recommendations. The consensus guideline of the American Psychosocial Oncology Society (APOS) and the Association of Oncology Social Work (AOSW) emphasizes the importance of fostering eHealth literacy through patient-centered communication and shared decision making to support informed choices even under psychological strain [36]. Similarly, the National Cancer Institute recommends that healthcare providers proactively address CAM use, explore patients’ underlying motivations and goals, and guide them toward evidence-based, quality-assured information sources [37].

Our findings suggest that CAM use may be associated more closely with individual needs, expectations, and healthcare experiences than with online forum use alone [38]. These findings should also be interpreted in light of previous international and European research showing that CAM use in oncology is frequently associated with female gender, higher educational level, and greater patient involvement in health-related decision-making. Large European surveys as well as studies from Sweden and Norway have reported comparable utilization patterns and motivations for CAM use among cancer patients [22–24]. People who use traditional, complementary, and integrative medicine (TCIM) because of a negative attitude toward conventional medicine are more likely to consider (online) media as their most important source of medical information, while the importance of medical professionals declines [39]. In prior research, dissatisfaction with conventional medicine, including perceived inadequate symptom control or insufficiently patient-centered communication, has been associated with greater interest in CAM approaches [20, 40, 41].

Digital information sources and social media may function as contextual or supportive influences rather than primary drivers in this context. As previous studies show, they primarily reflect an increased need for autonomy and information [42]. However, the present study did not find any correlation between eHealth literacy and more intensive use of complementary and alternative medicine, which underscores the distinction between information literacy and actual health behavior. Uncertainties regarding the quality and reliability of information available online and the spread of misinformation are discussed in the literature as potential influencing factors, but could not be directly proven in the present study [43].

The use of online forums by cancer patients varies significantly depending on the stage of the disease. Shortly after diagnosis, the need for information is particularly high, which is accompanied by increased consultation of digital information sources and online forums [17, 44]. As the duration of the illness increases, the use of digital services changes in that patients often obtain relevant medical information at an early stage, establish individually helpful coping or complementary strategies, and therefore use open online forums less frequently for the sole purpose of searching for information, while these forums increasingly take on functions of emotional support, exchange of coping strategies, and meaning-making [45, 46]. Accordingly, social interaction becomes more important as the disease progresses, while the information-oriented benefits decrease [45]. Direct exchange with other people in similar situations in peer support groups or self-help groups can reduce unrealistic expectations and is associated with positive effects on self-efficacy, health behavior, and eHealth literacy [47–49], although recommendations within such groups are not necessarily evidence-based [50, 51]. Against this background, it seems sensible to view online forums as a complementary support structure, ideally accompanied by medical supervision and focused on quality-assured, evidence-based content in order to avoid potentially problematic information and behavior patterns [50, 52, 53].

In summary, the findings indicate that the use of CAM is primarily influenced by individual needs, expectations, previous experiences with healthcare and other media, while forum-based digital resources appear to play only a secondary role. It is noteworthy that discussions about CAM in online forums were consistently rated as less trustworthy than those about conventional practices, regardless of eHealth literacy, psychological stress, or the role of the respondents. These findings should not be generalized to other digital environments such as social media platforms, which differ substantially from curated patient forums in structure, audience, and information dynamics. Online forums function less as primary drivers of CAM use and more as context-dependent, downstream resources whose function shifts over the course of an illness from initial information gathering to forms of emotional and social support. Overall, the results suggest a selective, needs-based usage model and underscore the importance of early, patient-centered medical support for digital information and decision-making processes.

## Limitations and future directions

This study has several limitations. Its cross‑sectional design precludes causal inference, and the reliance on self‑reported forum use introduces potential recall and reporting bias. Voluntary participation may have led to self‑selection, particularly among digitally experienced or health‑conscious individuals. In addition, cancer type, disease stage, and survivor status were self-reported and could not be independently verified, introducing the possibility of misclassification. Particularly regarding distinctions between localized and advanced disease stages, participants may have differed in their understanding or interpretation of clinical terminology. The dichotomization of forum exposure simplified modeling but reduced variability and may have obscured more nuanced associations. Limited statistical power further restricted the detection of smaller effects and contributed to wide confidence intervals.

Outcome and exposure measurement also present constraints. CAM use, CAM-related attitudes, and CAM-related decision-making were assessed using self-reported questionnaire measures. In addition, the broad range of CAM approaches included interventions with varying levels of evidence, which may have diluted associations within specific CAM categories. Forum exposure was operationalized solely through visit frequency, omitting dimensions such as session duration, membership length, or active participation. The lack of correlation between frequency and session duration (Spearman’s *ρ* = 0.07, *p* = 0.20) suggests that frequency alone insufficiently captures engagement depth and may have led to misclassification.

Despite these limitations, the study provides meaningful insights into digital engagement, psychological distress, and CAM endorsement within an online cancer community, offering an important foundation for future research.

## Conclusion

This study shows that the use of CAM in cancer care was not primarily driven by forum content. Importantly, the Krebs-Kompass forum examined in this study was moderatedto prevent commercial advertising and overtly problematic content, while allowing the discussion of non-biomedical and CAM-related perspectives. Accordingly, these conclusions apply to moderated online forums and should not be generalized to other digital platforms such as social media. Neither eHealth literacy nor psychological distress was associated with CAM use intensity, trust in forum-based discussions, or the perceived effectiveness of specific CAM modalities. Although patients used online cancer forums more frequently than non-patients, their trust in forum content and their CAM-related attitudes were largely comparable.

Across all subgroups, CAM-related forum discussions were consistently perceived as less trustworthy than discussions on conventional cancer treatments, indicating selective and differentiated trust judgments rather than uncritical acceptance of online information. Overall, CAM use appears to be primarily associated with individual needs, expectations, and prior healthcare experiences, while digital media play a secondary and context-dependent role. These findings highlight the importance of patient-centered communication and proactive clinical engagement with patients’ information-seeking behaviors to support informed and evidence-based decision-making.

## Supplementary Information

Below is the link to the electronic supplementary material.

ESM 1 (DOCX 33.6 KB)

ESM 2 (DOCX 28.8 KB)

ESM 3 (DOCX 41.6 KB)

## Data Availability

The datasets generated during and/or analysed during the current study are available from the corresponding author on reasonable request.
