# Supplementary material for: Online cancer forums and complementary and alternative medicine in cancer care: effects of ehealth literacy and psychological distress on use and attitudes
Source: Support Care Cancer. 2026 Aug 4;34(8):827. doi: 10.1007/s00520-026-11049-3 (PMC13437653; doi:10.1007/s00520-026-11049-3)
Supplement: Supplementary file 1 [file 520_2026_11049_MOESM1_ESM.docx]

Krebs-Kompass Survey

# Beginning of block: Introduction

**Q1.1 Purpose of the survey**

Thank you for taking the time to participate in this important survey. This survey is part of a scientific study conducted by Prof. Dr. Marcus Oehlrich, the founder of krebs-kompass.de. It examines whether and how online cancer forums such as Krebs-Kompass influence treatment decisions made by patients. The survey consists of 24 questions and takes approximately 6-7 minutes to complete. The survey is intended for patients as well as relatives and friends of patients.

In general, you can complete the questionnaire on any device (PC, tablet, smartphone). Some questions require text entry.

## Independence

I am a university lecturer and researcher and have been working on the topic of cancer information on the Internet for 25 years. The study is conducted solely out of scientific interest. There is no collaboration with companies, no third-party funding is provided, and no data will be shared with third parties. The findings from the study are intended to be published scientifically.

## Voluntary participation

You must be at least 18 years old to participate in this survey. Your participation is entirely voluntary. You may skip individual questions or discontinue your participation at any time.

## Anonymity, confidentiality, and language

Please do not provide your name or any other identifying information. All responses will be anonymized and analyzed only in aggregated form. For reasons of readability, all personal designations apply to all genders.

## Questions

If you have any questions or comments, you may contact marcus.oehlrich@krebs-kompass.de at any time.

To begin the survey, please click the "Next page" link at the bottom of this page. The link next to it allows you to return to previous pages.

# End of block: Introduction

# Beginning of block: About you

**Q2.1 How old are you?**

- 18 to 25 years (1)
- 26 to 35 years (2)
- 36 to 45 years (3)
- 46 to 55 years (4)
- 56 to 65 years (5)
- 66 to 75 years (6)
- 76 years or older (7)

**Q2.2 How would you describe your gender?**

- Male (1)
- Female (2)
- Diverse (3)
- Prefer not to say (4)

**Q2.3 What is your highest level of education?**

- No school-leaving certificate (1)
- Intermediate school-leaving certificate (e.g., Hauptschule, Realschule, lower secondary education, polytechnic school) (2)
- Vocational or university degree (e.g., apprenticeship, technical college, college, university studies) (3)
- Other (4)

**Q2.4 Please indicate the extent to which you agree with the following statements:**

| **Statement** | **Strongly disagree (1)** | **Partly disagree (2)** | **Neither agree nor disagree (3)** | **Partly agree (4)** | **Strongly agree (5)** |
| --- | --- | --- | --- | --- | --- |
| I know how to find useful health information on the Internet. (1) |  |  |  |  |  |
| I know how to use the Internet to get answers to my questions about health-related topics. (2) |  |  |  |  |  |
| I know which sources of health information are available on the Internet. (3) |  |  |  |  |  |
| I know where I can find useful health information on the Internet. (4) |  |  |  |  |  |
| I know how to use information from the Internet in a way that helps me. (5) |  |  |  |  |  |
| I am able to critically evaluate information that I find on the Internet. (6) |  |  |  |  |  |
| I can distinguish reliable information from questionable information on the Internet. (7) |  |  |  |  |  |
| When I make health-related decisions based on information from the Internet, I feel confident doing so. (8) |  |  |  |  |  |

# End of block: About you

# Beginning of block: Diagnosis and information behavior in cancer

**Q3.1 What is your role in relation to the cancer diagnosis?**

- I am a patient and am currently receiving treatment. (1)
- I had cancer and am currently cancer-free. (2)
- I am a relative or partner of a patient. (3)
- I am a close friend of a patient. (4)
- Other (please specify): (5) ________________________________________________

**Q3.2 Please answer the following questions from the perspective corresponding to the role you indicated:**

If you are personally affected (patient), please base your answers on your own experiences. If you are supporting an affected person (relative, partner, friend), please answer the questions from your perspective as an accompanying or supporting person.

**Q3.3 What type of cancer was diagnosed?**

- Pancreatic cancer (1)
- Bladder cancer (2)
- Breast cancer (3)
- Colorectal cancer (4)
- Skin cancer (5)
- Leukemia or lymphoma (6)
- Lung cancer (7)
- Prostate cancer (8)
- Other (please specify): (9) ________________________________________________
- I do not know (10)

**Q3.4 What is the current stage of the cancer?**

- Localized (confined to the site of origin) (1)
- Regional (spread to nearby tissue or lymph nodes) (2)
- Metastatic (spread to distant parts of the body) (3)
- I do not know (4)

**Q3.5 When was the cancer diagnosis made?**

- Less than 1 year ago (1)
- More than 1 to 2 years ago (2)
- More than 2 to 5 years ago (3)
- More than 5 to 10 years ago (4)
- More than 10 years ago (5)
- I do not know (6)

**Q3.6 Which treatments have been carried out in the last three months? (Multiple responses possible)**

- Surgery (1)
- Chemotherapy (2)
- Radiation therapy (3)
- Immunotherapy (4)
- Hormone therapy (5)
- Experimental therapies (e.g., participation in clinical trials) (6)
- Palliative care (7)
- Complementary or alternative medicine (8)
- Other (please specify): (9) ________________________________________________
- I do not know (10)

**Q3.7 How often have you been bothered by the following problems over the last two weeks? (Please select the appropriate answer for each statement.)**

| **Problem** | **Not at all (1)** | **Several days (2)** | **More than half the days (3)** | **Nearly every day (4)** |
| --- | --- | --- | --- | --- |
| Little interest or pleasure in your activities (1) |  |  |  |  |
| Feeling down, depressed, or hopeless (2) |  |  |  |  |
| Feeling nervous, anxious, or tense (3) |  |  |  |  |
| Being unable to relax or control worrying (4) |  |  |  |  |

# End of block: Diagnosis and information behavior in cancer

# Beginning of block: Patterns of online forum use

**Q4.1 How often do you visit online forums about cancer?**

- Very often (almost daily or several times per week) (1)
- Often (weekly, but not several times per week) (2)
- Sometimes (occasionally, but not weekly) (3)
- Rarely (only in specific or exceptional situations) (4)
- Never (5)

**Q4.2 Which online forums about cancer do you use? (Name or link/URL; please list all)**

________________________________________________________________

________________________________________________________________

________________________________________________________________

________________________________________________________________

________________________________________________________________

**Q4.3 How much time do you usually spend in these forums per visit?**

- Less than 15 minutes (1)
- 15-30 minutes (2)
- 31-60 minutes (3)
- More than 1 hour (4)
- I do not use online forums about cancer. (5)

**Q4.4 For what reasons do you use online forums about cancer? (Multiple responses possible. If you do not use online forums, please leave this question unanswered.)**

- Searching for information on specific treatments (1)
- Searching for treatments not previously known to me (2)
- Receiving emotional support (3)
- Sharing personal experiences (4)
- Contacting others in similar situations (5)
- Other (please specify): (6) ________________________________________________

**Q4.5 How do you mostly use online forums about cancer?**

- I regularly write posts and participate in discussions. (1)
- I occasionally write or comment. (2)
- I communicate mainly or only with other users by private message or email. (3)
- I only read along and do not actively participate. (4)
- I do not use online forums about cancer. (5)

**Q4.6 What factors would make you trust treatment recommendations from other forum users? (Multiple responses possible)**

- Similarity of the user's illness (1)
- Detail of the experience described (2)
- Several users report similar outcomes (3)
- Lack of alternative sources of information (4)
- Lack of trust in the treating physician (5)
- Other (please specify): (6) ________________________________________________

# End of block: Patterns of online forum use

# Beginning of block: Attitudes toward CAM

**Q5.1**

In addition to conventional treatment methods ("conventional medicine"), such as surgery, chemotherapy, or radiation therapy, there are complementary and alternative medicine procedures for cancer treatment. This term includes a wide range of procedures that cancer patients use in addition to conventional treatments (complementary) or instead of them (alternative). For some of these procedures, scientific evidence regarding their effectiveness and/or safety is available, while for others such evidence is lacking - or it has even been scientifically demonstrated that they are ineffective and/or harmful. Regardless of your attitude toward these procedures or whether you have already used them, please answer these final questions.

**Q5.2 Do you personally consider one or more of the following complementary and alternative medicine procedures for cancer treatment to be promising? (Multiple responses possible. If you do not consider any of these procedures promising, please leave this question unanswered.)**

- Herbal remedies (e.g., aloe vera, green tea, manuka honey, but not mistletoe) (1)
- Dietary supplements (e.g., vitamins, minerals) (2)
- Acupuncture (including acupressure) (3)
- Homeopathy (e.g., globules, Schuessler salts) (4)
- Cancer diets (e.g., ketogenic diet, fasting cures) (5)
- Anthroposophic medicine (e.g., mistletoe therapy) (6)
- Massage therapy (e.g., classical massage, shiatsu) (7)
- German New Medicine according to Hamer (8)
- Other (please specify): (9) ________________________________________________

**Q5.3 How confident are you in distinguishing between conventional treatments and complementary and alternative medicine procedures?**

- Very confident (1)
- Rather confident (2)
- Neutral (3)
- Rather unsure (4)
- Not confident at all (5)

**Q5.4 How trustworthy do you find discussions in online forums about the effectiveness and safety of complementary and alternative medicine procedures?**

- Very trustworthy (1)
- Rather trustworthy (2)
- Neutral (3)
- Rather not trustworthy (4)
- Not trustworthy at all (5)
- I do not know (6)

**Q5.5 In comparison: How trustworthy do you find discussions in online forums about the effectiveness and safety of conventional procedures for cancer treatment?**

- Very trustworthy (1)
- Rather trustworthy (2)
- Neutral (3)
- Rather not trustworthy (4)
- Not trustworthy at all (5)
- I do not know (6)

**Q5.6 How confident are you in the accuracy of information provided in online forums about complementary and alternative medicine procedures for cancer treatment?**

- Very confident (1)
- Rather confident (2)
- Neutral (3)
- Rather unsure (4)
- Not confident at all (5)
- I do not know (6)

**Q5.7 How often have you read posts in online forums about treatments that are not scientifically proven in the last three months?**

- Very often (almost daily or several times per week) (1)
- Often (weekly, but not several times per week) (2)
- Sometimes (occasionally, but less than once per week) (3)
- Rarely (only in specific or exceptional situations) (4)
- Never (5)

**Q5.8 How strongly do forum discussions influence your willingness to consider treatments that are not scientifically proven for yourself or to recommend them to the patient?**

- Very strongly (1)
- Strongly (2)
- Moderately (3)
- Slightly (4)
- Not at all (5)

**Q5.9 Which aspects of forum discussions could make you consider treatments that are not scientifically proven for yourself or recommend them to the patient? (Multiple responses possible)**

- Positive experience reports from users (1)
- Lack of other treatment options (2)
- Emotional encouragement from the community (3)
- Links to scientific studies or external sources (4)
- Perceived safety of the treatment (5)
- Other (please specify): (6) ________________________________________________
- None (7)

# End of block: Attitudes toward CAM
