# Supplementary material for: Online cancer forums and complementary and alternative medicine in cancer care: effects of ehealth literacy and psychological distress on use and attitudes
Source: Support Care Cancer. 2026 Aug 4;34(8):827. doi: 10.1007/s00520-026-11049-3 (PMC13437653; doi:10.1007/s00520-026-11049-3)
Supplement: Supplementary file 2 [file 520_2026_11049_MOESM2_ESM.docx]

# STROBE Checklist for Cross-Sectional Studies

Manuscript: “Online Cancer Forums and Complementary and Alternative Medicine in Cancer Care: Effects of eHealth Literacy and Psychological Distress on Use and Attitudes”

| Item | Recommendation | Reported on page(s) |
| --- | --- | --- |
| 1(a) | Indicate the study’s design in the title or abstract | Title page, Abstract (pp. 1, 4) |
| 1(b) | Provide an informative and balanced summary in the abstract | Abstract (pp. 4–5) |
| 2 | Explain the scientific background and rationale | Introduction (pp. 5–7) |
| 3 | State specific objectives and hypotheses | Introduction (p. 7) |
| 4 | Present key elements of study design early in the paper | Patients and Methods – Study population (p. 8) |
| 5 | Describe setting, locations, and relevant dates | Study population (p. 8) |
| 6(a) | Describe eligibility criteria and participant selection | Inclusion and exclusion criteria (pp. 9–10) |
| 7 | Clearly define outcomes, exposures, predictors, confounders | Endpoints; Questionnaire (pp. 10–13) |
| 8* | Describe data sources and methods of assessment | Questionnaire (pp. 10–13) |
| 9 | Describe efforts to address potential sources of bias | Limitations and Future Directions (pp. 32–33) |
| 10 | Explain how study size was arrived at | Study population; Results (pp. 8, 15) |
| 11 | Explain handling of quantitative variables | Statistical analysis (pp. 13–14) |
| 12(a) | Describe all statistical methods | Statistical analysis (pp. 13–14) |
| 12(b) | Describe methods for subgroup analyses/interactions | Statistical analysis; Results (pp. 13–14, 19–29) |
| 12(c) | Explain how missing data were addressed | Questionnaire; Statistical analysis (pp. 13–14) |
| 12(d) | Describe analytical methods accounting for sampling strategy | Not applicable |
| 12(e) | Describe sensitivity analyses | Not performed |
| 13(a) | Report numbers at each study stage | Figure 1; Study population (pp. 8–9) |
| 13(b) | Give reasons for non-participation | Study population (p. 8) |
| 13(c) | Consider use of a flow diagram | Figure 1 (p. 9) |
| 14(a) | Give characteristics of study participants | Results – Demographic Data; Table 1 (pp. 15–18) |
| 14(b) | Indicate missing data for each variable | Table 1; Questionnaire (pp. 13, 16–18) |
| 15 | Report numbers of outcome events or summary measures | Results (pp. 19–29) |
| 16(a) | Give unadjusted and adjusted estimates with precision | Results (pp. 20–29) |
| 16(b) | Report category boundaries when continuous variables categorized | Questionnaire; Statistical analysis (pp. 12–14) |
| 16(c) | Translate estimates into absolute risk if relevant | Not applicable |
| 17 | Report other analyses performed | Results (pp. 19–29) |
| 18 | Summarize key results with reference to objectives | Discussion (pp. 29–32) |
| 19 | Discuss limitations including bias/imprecision | Limitations and Future Directions (pp. 32–33) |
| 20 | Provide cautious interpretation of results | Discussion (pp. 29–32) |
| 21 | Discuss generalizability | Discussion; Conclusion (pp. 31–34) |
| 22 | Give funding source and role of funders | Funding statement (p. 35) |
