# Supplementary material for: Online cancer forums and complementary and alternative medicine in cancer care: effects of ehealth literacy and psychological distress on use and attitudes
Source: Support Care Cancer. 2026 Aug 4;34(8):827. doi: 10.1007/s00520-026-11049-3 (PMC13437653; doi:10.1007/s00520-026-11049-3)
Supplement: Supplementary file 3 [file 520_2026_11049_MOESM3_ESM.docx]

# Supplementary Material

Article title: Online Cancer Forums and Complementary and Alternative Medicine in Cancer Care: Effects of eHealth Literacy and Psychological Distress on Use and Attitudes

Journal: Journal of Cancer Survivorship

Authors: G. Ciarlo, M. Oehlrich, J. Huebner

Corresponding author: Dr. med. Gianluca Ciarlo

Affiliation: University Hospital Frankfurt, Germany

E-mail: Gianluca.Ciarlo@unimedizin-ffm.de

## Supplementary Figure S1

High-exposure rates over the course of cancer survivorship

High exposure {%]

|  | High exposure |
| --- | --- |
| Less than 1 year | 36.6 |
| More than 1-2 years | 22.1 |
| More than 2-5 years | 28.8 |
| More than 5-10 years | 28.1 |
| Over 10 years | 12.9 |

Note. High exposure patients included patients who visited online forums weekly or several times a week.

## Supplementary Table S1

Item-level descriptive statistics of the eHealth Literacy scale

| **Item** | **M** | **SD** | **n** |
| --- | --- | --- | --- |
| I know how to find helpful health resources on the Internet *(1 = strongly disagree to 5 = strongly agree)* | 4.45 | 0.72 | 445 |
| I know how to use the Internet to answer my health questions *(1–5)* | 4.50 | 0.69 | 445 |
| I know what health resources are available on the Internet *(1–5)* | 4.28 | 0.78 | 445 |
| I know where to find helpful health resources on the Internet *(1–5)* | 4.41 | 0.73 | 445 |
| I know how to use the health information I find on the Internet *(1–5)* | 4.32 | 0.76 | 445 |
| I have the skills I need to evaluate the health resources I find on the Internet *(1–5)* | 4.29 | 0.79 | 445 |
| I can distinguish between high-quality and low-quality health resources on the Internet *(1–5)* | 4.34 | 0.77 | 445 |
| I feel confident using online health information to make health-related decisions *(1–5)* | 3.59 | 1.01 | 445 |

Note. Higher values indicate higher self-reported eHealth literacy.

## Supplementary Table S2

Item-level descriptive statistics of the Psychological Distress scale

| **Item** | **% ≥ “more than half the days”** | **M** | **SD** | **n** |
| --- | --- | --- | --- | --- |
| Little interest or pleasure in doing things *(1 = not at all to 4 = nearly every day)* | 26 % | 2.98 | 1.09 | 401 |
| Feeling down, depressed, or hopeless *(1–4)* | 24 % | 2.87 | 1.05 | 401 |
| Feeling nervous, anxious, or on edge *(1–4)* | 31 % | 3.15 | 1.01 | 401 |
| Not being able to stop or control worrying (1–4) | 29 % | 3.23 | 0.98 | 401 |

Note. Items were rated on a 4-point Likert scale ranging from 1 = not at all to 4 = nearly every day. Higher values indicate greater psychological distress.
